# A Species-Specific Anti-Human P2X7 Monoclonal Antibody Reduces Graft-versus-Host Disease in Humanised Mice

**DOI:** 10.3390/pharmaceutics15092263

**Published:** 2023-08-31

**Authors:** Amal Elhage, Peter Cuthbertson, Chloe Sligar, Debbie Watson, Ronald Sluyter

**Affiliations:** 1 Molecular Horizons and School of Chemistry and Molecular Bioscience, University of Wollongong, Wollongong, NSW 2522, Australia; ae880@uowmail.edu.au (A.E.); cpeter@uow.edu.au (P.C.); cs821@uowmail.edu.au (C.S.); dwatson@uow.edu.au (D.W.); 2 Illawarra Health and Medical Research Institute, Wollongong, NSW 2522, Australia

**Keywords:** xenogeneic graft-versus-host disease, P2X7, P2RX7, purinergic signalling, therapeutic antibody, biologic, regulatory T cells, T helper 17 cells, natural killer cells, natural killer T cells

## Abstract

Graft-versus-host disease (GVHD) is a T cell-mediated inflammatory disorder that arises from allogeneic haematopoietic stem cell transplantation and is often fatal. The P2X7 receptor is an extracellular adenosine 5′-triphosphate-gated cation channel expressed on immune cells. Blockade of this receptor with small molecule inhibitors impairs GVHD in a humanised mouse model. A species-specific blocking monoclonal antibody (mAb) (clone L4) for human P2X7 is available, affording the opportunity to determine whether donor (human) P2X7 contributes to the development of GVHD in humanised mice. Using flow cytometric assays of human RPMI 8266 and murine J774 cells, this study confirmed that this mAb bound and impaired human P2X7. Furthermore, this mAb prevented the loss of human regulatory T cells (hTregs) and natural killer (hNK) T cells in vitro. NOD-*scid* IL2Rγ^null^ mice were injected with 10 × 10^6^ human peripheral blood mononuclear cells (Day 0) and an anti-hP2X7 or control mAb (100 μg i.p. per mouse, Days 0, 2, 4, 6, and 8). The anti-hP2X7 mAb increased hTregs and hNK cells at Day 21. Moreover, anti-hP2X7 mAb-treatment reduced clinical and histological GVHD in the liver and lung compared to the control treatment at disease endpoint. hTregs, hNK, and hNK T cell proportions were increased, and human T helper 17 cell proportions were decreased at endpoint. These studies indicate that blockade of human (donor) P2X7 reduces GVHD development in humanised mice, providing the first direct evidence of a role for donor P2X7 in GVHD.

## 1. Introduction

Allogeneic haematopoietic stem cell transplantation (HSCT) is a current therapeutic measure for blood cancers including leukaemia and lymphoma [1]. Graft-versus-host disease (GVHD) is a major problem arising from allogeneic HSCT. GVHD is characterised as a severe inflammatory response that results in the destruction of tissue in the liver, lung, skin, and gastrointestinal tract and is fatal in up to 35% of cases [2]. GVHD occurs when donor (graft) immune cells recognise the recipient (host) tissue as foreign, thereby causing an immune reaction. This immune reaction includes the activation of antigen presenting cells (APCs) and CD4^+^ and CD8^+^ T cells [3]. This process then leads to the release of pro-inflammatory cytokines including interferon (IFN) γ, interleukin (IL)-6, and IL-17, which cause tissue damage. These pro-inflammatory cytokines also activate APCs and T cells to create a positive feedback loop, ultimately resulting in further host tissue destruction [4]. Current prophylactic treatments for GVHD include various immune suppressants and/or post-transplant cyclophosphamide [5]. However, given the high mortality rate for GVHD, new therapies for this disease are required.

P2X7 is a trimeric ligand-gated ion channel expressed on APCs, natural killer (NK) cells, NK T cells, and T and B cells [6,7]. P2X7 is activated by extracellular adenosine 5′-triphosphate (ATP), a damage associated molecular pattern that is released following cell activation, damage, or death [8]. P2X7 activation induces a variety of pro-inflammatory effects in numerous cell types [9], some of which play a role in inflammatory diseases including GVHD [10,11]. Activation of this receptor can deplete immune suppressing regulatory T cells (Tregs) [12], a cell type that can limit GVHD progression. Furthermore, stimulation of P2X7 can induce T helper (Th) 17 cell differentiation [13], a cell type thought to contribute to GVHD severity [14], and can also assist the conversion of Tregs to Th17 cells [15]. With this, P2X7 can serve as a possible therapeutic target in GVHD development.

The role of P2X7 in GVHD pathophysiology and progression has previously been demonstrated in both allogeneic [16,17,18,19] and humanised [20,21,22] mouse models of GVHD using small molecular antagonists. Monoclonal antibodies (mAb) that block P2X7 activation provide an alternate approach to prevent GVHD, but the use of such mAbs are limited to a single study of P2X7 in a mouse model of colitis, which used an anti-mouse (m) P2X7 mAb [23]. The use of an anti-human (h) P2X7 mAb has yet to be examined in any animal model or in humans. In 1998, Buell and colleagues developed the first available mouse anti-hP2X7 mAb [24]. This mAb was shown to bind human but not mouse P2X7 and to block human P2X7 activity in vitro. Use of this mAb in in vitro assays is limited and it remains unknown if this mAb can block human P2X7 in vivo to prevent disease such as GVHD. A humanised mouse model that has been extensively used to study and test therapies in GVHD [25] affords the opportunity to examine this mAb in vivo. Moreover, this model provides an opportunity to explore the role of donor (human) P2X7 using this species-specific mAb in GVHD progression.

The current study confirmed the ability of the anti-hP2X7 mAb to bind and block human but not mouse P2X7 in vitro. Furthermore, the anti-hP2X7 mAb prevented the loss of hTregs and hNK T cells in a serum-reduced culture, which promotes cell death. Finally, administration of the anti-hP2X7 mAb in humanised mice reduced both clinical and histological GVHD, which corresponded with increased proportions of hTregs, hNK, and hNK T cells and a reduction in hTh17 cells.

## 2. Materials and Methods

### 2.1. Cell Lines

Human multiple myeloma RPMI 8226 cells were obtained from the European Collection of Cell Cultures (Salisbury, UK). Mouse macrophage J774 cells were obtained from American Type Culture Collection (Rockville, VA, USA). Both cell lines were maintained in RPMI-1640 medium (Thermo Fisher Scientific, Waltham, MA, USA) containing 10% foetal calf serum (FCS) (Bovogen, Keilor East, Australia) and 2 mM GlutaMAX (Thermo Fisher Scientific). The anti-hP2X7 mAb producing mouse hybridoma cell line (clone L4) was originally obtained from the Glaxo Institute for Applied Pharmacology (Cambridge, UK) and maintained in Iscove’s Modified Dulbecco’s Medium (IMDM) (Sigma Aldrich, St Louis, MO, USA) containing 20% FCS and 2 mM GlutaMAX. A mouse multiple myeloma cell line producing IgG2_b_ isotype control mAb (clone MPC-11) was obtained from CellBank Australia (Westmead, Australia) and maintained in Dulbecco’s Modified Eagle Medium (DMEM) (Sigma Aldrich) containing 20% horse serum (Sigma Aldrich) and 2 mM GlutaMAX. Cell lines were assessed for *Mycoplasma* spp. contamination using the Myco Alert Mycoplasma Detection Kit (Lonza, Basel, Switzerland) as per the manufacturer’s instructions and were found to be routinely negative. 

### 2.2. Purification and Conjugation of the Anti-hP2X7 and Isotype Control mAbs

The anti-hP2X7 mAb and mouse IgG2_b_ isotype control mAb was purified and conjugated to DyLight488 as described [26]. Briefly, L4 and MPC-11 cells were cultured in IMDM or DMEM, respectively, containing 5% serum until the majority of cells appeared dead. The resulting tissue culture supernatants (TCSNs) were collected. IgG from TCSNs was then purified using a Pierce Protein A Agarose IgG Purification Kit (Thermo Fisher Scientific) according to the manufacturer’s instructions. Briefly, TCSN was incubated with 1 mL Protein A resin for 90 min with gentle rocking. The resin–TCSN mixture was packed into a supplied chromatography column to form a bed. The sample was then allowed to flow through the column. The column was washed with the supplied binding buffer, and 1 mL fractions were collected. The absorbances of the fractions were measured at 280 nm using a Suprasil quartz cuvette (Hellma, Müllheim, Germany) in a SpectraMax Microplate Reader spectrophotometer (Molecular Devices, San Jose, CA, USA) to confirm that all unbound protein was removed from the column. Bound IgG was then eluted with the supplied elution buffer, with 1 mL fractions collected into microfuge tubes containing 50 μL of 1 M Tris solution (pH 9.5) and the absorbances measured as above. Fractions with the highest absorbances, indicating the elution of IgG, were pooled and concentrated to 1 mg/mL in Dulbecco’s phosphate-buffered saline (PBS) (Thermo Fisher Scientific), using a 50 kD molecular cut-off centrifugal device (Thermo Fisher Scientific) as per the manufacturer’s instructions for subsequent studies or for conjugating to DyLight488. 

Purified anti-hP2X7 and isotype control IgG were conjugated to DyLight488 using a DyLight Microscale Antibody Labelling Kit (Thermo Fisher Scientific) according to the manufacturer’s instructions. Briefly, 100 µL of mAb was incubated in the provided vial of DyLight488 with 8 µL of borate buffer for 60 min. During this incubation, 100 µL of purification resin was added to the provided spin column and centrifuged (1000× *g* for 1 min) to remove the storage solution and create a resin bed. Following incubation, the labelling mixture was added to the spin column and mixed with resin and centrifuged (1000× *g* for 1 min). The absorbance of the resulting flow through material containing the DyLight488-conjugated mAb was measured as above at 280 nm and 493 nm. Protein concentration and dye-to-protein ratio was calculated using the provided formulae, and routinely yielded a ratio of 1.6.

### 2.3. P2X7 Expression by Immunolabelling and Flow Cytometry

Human RPMI 8226 and mouse J774 cells were washed in PBS containing 10% FCS (300× *g* for 5 min). Cells (1 × 10^6^ cells/100 µL) were incubated with DyLight488-conjugated anti-hP2X7 or isotype control mAbs, or phycoerythrin (PE)-conjugated anti-mP2X7 (clone 1F11) (BioLegend, San Diego, CA, USA) or rat IgG2_b_ isotype control (clone RTK4530) (BioLegend) mAbs and the cell viability dye 7-aminoactinomycin D (7AAD) (1 µg/mL) (Enzo Life Sciences, Farmingdale, NY, USA) for 20 min in the dark. Cells were washed and resuspended in PBS and data acquired with a BD Biosciences (San Diego, CA, USA) LSR Fortessa X-20 flow cytometer using an excitation wavelength of 488 nm for DyLight488 and 7AAD, and 561 nm for PE, and detection wavelengths of 525/50, 586/15, and 675/20 nm for DyLight488, PE, and 7AAD, respectively. The mean fluorescence intensity (MFI) of mAb labelling was determined using the geometric mean function of FlowJo software v10.7.1 (BD Biosciences). The relative P2X7 expression on cells was determined as the difference between the MFI of anti-human or anti-mouse P2X7 mAb and the corresponding isotype control mAb. 

### 2.4. ATP-Induced YO-PRO-1^2+^ Dye Uptake Assay

The P2X7 activity in cells was assessed using an ATP-induced uptake of YO-PRO-1^2+^ as described [27]. Human RPMI 8226 and mouse J774 cells were washed and resuspended in low divalent medium (LDM) (145 mM NaCl, 5 mM KCl, 0.2 mM CaCl_2,_ 13 mM glucose, and 10 mM HEPES, pH 7.5) (300× *g* for 5 min) (1 × 10^6^ cells/mL). Cells were pre-incubated for 5 min at 37 °C and then with 1 µM YO-PRO-1 iodide (Invitrogen, Carlsbad, CA, USA) for another 5 min at 37 °C with or without ATP. Incubations were terminated by the addition of an equal volume of ice-cold LDM containing 20 mM MgCl_2_ and centrifugation (300× *g* for 3 min). Cells were washed once with LDM (300× *g* for 3 min) and resuspended in LDM for flow cytometric analysis. For assays using mAbs, cells were pre-incubated with mAbs or PBS for 10 min at 37 °C prior to the addition of YO-PRO-1 iodide and ATP. Data were acquired with a BD Biosciences Accuri flow cytometer using an excitation wavelength of 488 nm and a detection wavelength of 525/50 nm. The MFI of YO-PRO-1^2+^ uptake was determined using the geometric mean function of the FlowJo v10.7.1 software. Data were normalised to percent of control (PBS in the presence of ATP), which was set as 100%. YO-PRO-1^2+^ uptake in the presence of anti-hP2X7 or isotype mAb was calculated as a percent of control YO-PRO-1^2+^ uptake.

### 2.5. Human Peripheral Blood Mononuclear Cell Isolation 

Human blood was collected and utilised in accordance with approval by the University of Wollongong Human Ethics Committee (HE 12/290). Human peripheral blood mononuclear cells (hPBMCs) were isolated as described [28]. Briefly, whole blood was collected into Vacutainer heparin tubes (BD Biosciences) from healthy donors (six males; three females; age range 23–50 years) and diluted in an equivalent volume of sterile PBS. Samples were underlaid with Ficoll-Paque^TM^ PLUS (GE Healthcare; Uppsala, Sweden) and centrifuged (560× *g* for 30 min with brake disengaged). hPBMCs were recovered from the gradient interface and washed twice with PBS and resuspended at a final concentration of 5 × 10^6^ cells/mL in RPMI-1640 medium containing 40% FCS for cryopreservation as described [29] or 10 × 10^7^ cells/mL in PBS for injection into mice. 

### 2.6. Human Peripheral Blood Mononuclear Cell In Vitro Culture 

Cryopreserved hPBMCs were thawed and studied as described [21]. Briefly, hPBMCs were washed with PBS, then twice with RPMI-1640 medium containing 2.5% FCS and 2 mM GlutaMAX (300× *g* for 5 min). hPBMCS were plated (2 × 10^6^ cells/well of flat-bottomed 24-well plates) in medium and incubated with sterile-filtered anti-hP2X7 or isotype control mAb (2 µg/mL) for 20 h at 37 °C (95% air/5% CO_2_). hPBMCs from the wells were transferred to tubes and washed with PBS (300× *g* for 5 min) and analysed by flow cytometry, as outlined below (Section 2.9). 

### 2.7. Humanised Mouse Model of Graft-Versus-Host Disease

All animal experiments were conducted in accordance with approval by the University of Wollongong Animal Ethics Committee (AE 18/05). A humanised mouse model of GVHD was used as described [28]. Female NOD-*scid* IL2Rγ^null^ (NSG) mice (aged 6–8 weeks) from the Animal Resources Centre (Canning Vale, Australia) were acclimatised for two weeks and injected intraperitoneally (i.p.) with 10 × 10^6^ freshly isolated hPBMCs. Mice were subsequently injected i.p. with either sterile-filtered anti-hP2X7 or isotype control mAb (100 µg/mouse) 2 h post-hPBMC injection (Day 0), then every second day (Days 2, 4, 6, and 8). Mice were monitored, in a blinded fashion, thrice weekly for weight loss, survival, and clinical score until endpoint using an established scoring system [28]. Ear thickness was measured once weekly until disease onset (Day 21), then three times a week until humane or ethical endpoint using Interapid spring-loaded callipers (Rolle, Switzerland). Mice were euthanised at humane (disease) endpoint or experimental endpoint (Day 21 or 75) by slow-fill CO_2_ and organs were obtained for analyses. 

### 2.8. Histological Analyses

Organs were removed from euthanised mice and processed as described [30]. Briefly, samples were fixed with neutral buffered (10%) formalin (Sigma-Aldrich), embedded in paraffin wax, sectioned (3–5 μm) using an RM2255 microtome (Leica Biosystems; Wetzlar, Germany) and stained with haematoxylin and eosin (POCD, Atarmon, Australia). Histological differences were measured using a Leica DMIL inverted light microscope with a 20× objective. GVHD in the liver, skin, and ear was assessed, in a blinded fashion, using a standardised grading system (using grades from 0 to 4) as described [28]. GVHD in the lung was assessed, in a blinded fashion, as the percent clear alveoli space through Fiji software of the total lung area measured as described [30].

### 2.9. Immunophenotyping by Flow Cytometry

Spleens and livers from humanised mice were collected, dissociated, and immunophenotyped as described [21]. Briefly, homogenates were filtered through a 70 μm nylon filter (BD Bioscience) and centrifuged (300× *g* for 5 min) before being incubated for 5 min with red cell lysis buffer (150 mM NH_4_Cl, 1 mM KHCO_3_, 0.1 mM Na_2_CO_3_). hPBMCs were washed and resuspended in PBS (1 × 10^6^ cells/tube). Cells were stained with Zombie Near Infrared live/dead dye (BioLegend) for 15 min in the dark on ice. Cells were subsequently washed with PBS containing 2% FCS (300× *g* for 5 min). Cells were incubated with fluorochrome-conjugated mAbs (Table S1) for 15 min in the dark on ice. Samples were washed (300× *g* for 5 min) and resuspended in PBS and data were acquired using a BD Biosciences LSR Fortessa X-20. Proportions of immune cell subsets were assessed using FlowJo software version 10.7.1. 

### 2.10. Serum Cytokine Analyses

Serum was collected from euthanised mice as described [21]. Cytokine concentrations were determined using a human T Helper-1 LEGENDplex Kit (BioLegend) as per the manufacturer’s instructions. Human IFNγ concentrations were measured using a human IFNγ ELISA Kit (Thermo Fisher Scientific) as per the manufacturer’s instructions with a SpectraMax Plus 384 plate reader. 

### 2.11. Data Presentation and Statistical Analyses

Data are represented as the mean ± standard error of the mean (SEM). Data were tested for normality using a Shapiro–Wilk test. Statistical differences were calculated using an unpaired (or paired as indicated) Student’s *t*-test (two-tailed) (parametric) or Mann–Whitney test (non-parametric) for single comparisons or one-way analysis of variance (ANOVA) with a Tukey’s post-hoc test for multiple comparisons. Weight, clinical score, and ear thickness differences in the mice were determined with a two-way ANOVA with the Bonferroni post-hoc test, with data from euthanised mice carried forward. Survival differences were determined by a log-rank (Mantel–Cox) test. All statistical analyses were conducted and graphs assembled using GraphPad Prism software v8.0.2 (GraphPad Software; La Jolla, CA, USA). For all analyses, differences were considered significant if *p* < 0.05.

## 3. Results

### 3.1. The Anti-hP2X7 mAb Binds and Blocks Human but Not Mouse P2X7 In Vitro

**Figure 1:**
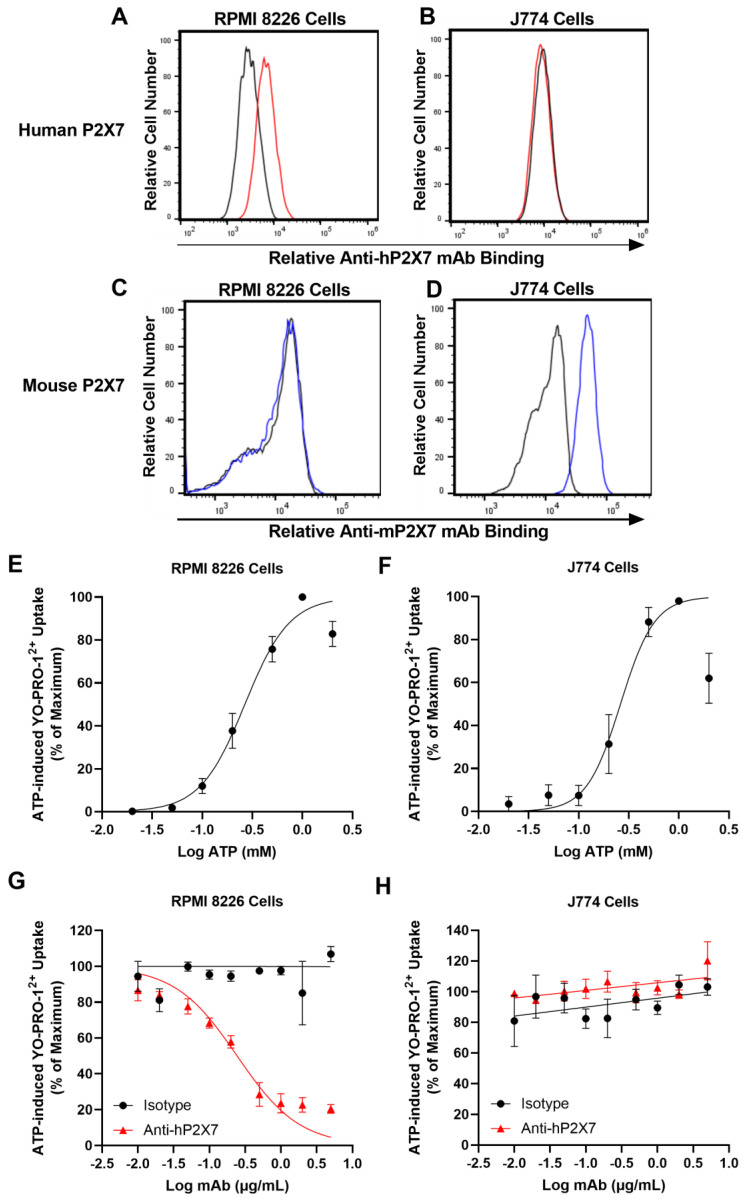
The anti-hP2X7 mAb binds and blocks human but not mouse P2X7. (**A**,**C**) Human RPMI 8226 and (**B**,**D**) mouse J774 cells were incubated with (**A**,**B**) DyLight488 conjugated anti-hP2X7 (red line) or isotype control (black line) mAb or (**C**,**D**) PE-conjugated anti-mP2X7 (blue line) or isotype control (black line) mAb and analysed by flow cytometry. (**E**) Human RPMI 8226 (*n* = 4) and (**F**) mouse J774 cells (*n* = 4) in LDM containing 1 µM YO-PRO-1^2+^ were incubated with 0.02 to 2 mM of ATP at 37 °C for 5 min. (**G**) Human RPMI 8226 (*n* = 3) and (**H**) mouse J774 cells (*n* = 3) in LDM were pre-incubated with 0.01 µg/mL to 5 µg/mL of the anti-hP2X7 or isotype control mAb at 37 °C for 10 min. Cells were then incubated with 1 µM of YO-PRO-1^2+^ in the absence or presence of 0.3 mM and 0.26 mM ATP for human RPMI 8226 and mouse J774 cells, respectively, at 37 °C for a further 5 min. (**E**–**H**) Incubations were terminated by the addition of ice-cold medium containing MgCl_2_ and centrifugation. YO-PRO-1^2+^ uptake was then analysed by flow cytometry and data normalised to the maximum ATP response in each experiment. (**E**–**H**) Results are the mean ± SEM.

Before testing the anti-hP2X7 mAb in vivo, the anti-hP2X7 mAb was tested in vitro to confirm its ability to bind and block human and not mouse P2X7 [24]. Both human RPMI 8226 cells [31] and mouse J774 cells [32] express functional cell-surface P2X7. To confirm the species-specificity of the anti-hP2X7 mAb, RPMI 8226 and J774 cells were incubated with DyLight488-conjugated anti-hP2X7 and isotype control mAb. To confirm the presence of mouse P2X7 on J774 cells, cells were also incubated with PE-conjugated anti-mP2X7 (clone 1F11) [23] and isotype control mAb. The anti-hP2X7 mAb showed a right shift in fluorescence for human RPMI 8226 cells (Figure 1A) but not mouse J774 cells (Figure 1B) compared to the corresponding isotype control. Conversely, the anti-mP2X7 mAb showed a right shift in fluorescence for mouse J774 cells (Figure 1D) but not human RPMI 8226 cells (Figure 1C) compared to the corresponding isotype control. MFI expression of hP2X7 or mP2X7 on RPMI 8226 and J774 cells was 1151 ± 286 and 34,531 ± 1059, respectively. Conversely, MFI values for anti-hP2X7 mAb on J774 cells and anti-mP2X7 mAb in RPMI 8226 cells were like that of the isotype control values.

To confirm that human RPMI 8226 and mouse J774 cells express functional P2X7 and to determine the half maximal effective concentration (EC_50_) for ATP, cells were incubated with YO-PRO-1^2+^ and increasing concentrations of ATP. ATP-induced YO-PRO-1^2+^ uptake in a concentration-dependent manner, with maximal uptake occurring at 1 mM ATP for the human RPMI 8226 cells (Figure 1E) and 1 mM ATP for mouse J774 cells (Figure 1F). The EC_50_ values for ATP were 0.28 ± 0.04 mM for human RPMI 8226 cells and 0.26 ± 0.02 mM for mouse J774 cells.

To confirm species-specificity of the anti-hP2X7 mAb, human RPMI 8226 and mouse J774 cells were pre-incubated with increasing concentrations of the anti-hP2X7 mAb and then incubated with ATP at the approximate EC_50_ values obtained above. The anti-hP2X7 mAb inhibited ATP-induced uptake of YO-PRO-1^2+^ into human RPMI 8226 cells in a concentration-dependent manner with maximum blockade (~85% inhibition) at 1 μg/mL and with a half maximum inhibitory concentration (IC_50_) of 0.21 ± 0.04 μg/mL (Figure 1G). Conversely, this mAb at any of the concentrations examined did not block ATP-induced YO-PRO-1^2+^ uptake into mouse J774 cells (Figure 1H). Likewise, the isotype control showed minimal to no blockade of ATP-induced YO-PRO-1^2+^ uptake into either human RPMI 8226 (Figure 1G) or mouse J774 cells (Figure 1H). Collectively, the expression and functional studies confirm the species-specificity of the anti-hP2X7 mAb. 

### 3.2. The Anti-hP2X7 mAb Prevents the Loss of hTregs and hNK T Cells In Vitro 

**Figure 2:**
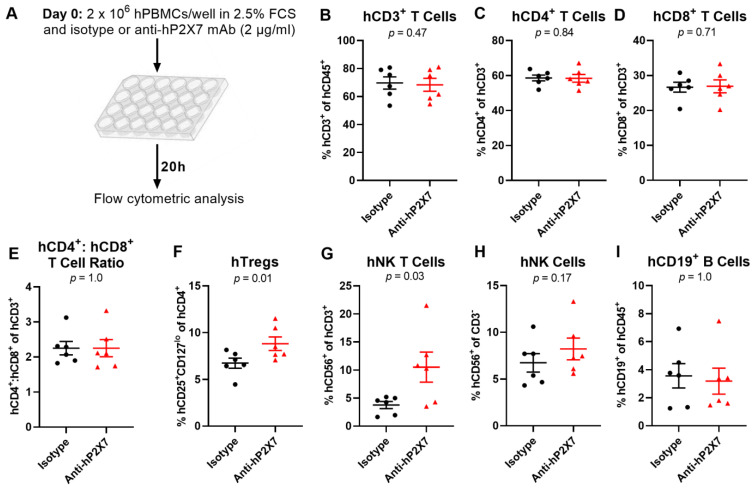
The anti-hP2X7 mAb prevents the loss of hTregs and hNK T cells in vitro. (**A**) Illustration of the in vitro cultures. (**B**–**I**) hPBMCs (*n* = 6 donors) were cultured overnight (20 h) in RPMI-1640 medium containing 2.5% FCS in the presence of 2 µg/mL of the isotype control or anti-hP2X7 mAb. The proportions of (**B**) hCD3^+^ T cells, (**C**) hCD4^+^ and (**D**) hCD8^+^ T cell subsets, (**F**) hCD4^+^hCD25^+^hCD127^lo^ hTregs, (**G**) hCD56^+^ hCD3^+^ hNK T cells, (**H**) hCD56^+^ hCD3^−^ hNK cells, or (**I**) CD19^+^ B cells were determined by flow cytometry. (**E**) The ratio of hCD4^+^ to hCD8^+^ T cells was calculated from (**C**,**D**). (**B**–**I**) Data represented as mean ± SEM, symbols represent individual donors. Significance was assessed by the paired Student’s *t*-test, with *p* values as shown.

Our previous study showed that culturing hPBMCs in low concentrations of FCS (<10%) results in cell death and that the P2X7 antagonist Brilliant Blue G (BBG) prevents the loss of Tregs, which was attributed to blockade of the P2X7-induced death of Tregs [21]. To determine whether the anti-hP2X7 mAb could induce the same effect as BBG, hPBMCs from six donors were cultured in 2.5% FCS in the presence of 2 µg/µL of the anti-hP2X7 or isotype control mAb (Figure 2A). Flow cytometric analysis, using the consistent gating strategy demonstrated in Figure S1, showed that the proportions of hCD3^+^ (*p* = 0.47), hCD4^+^ (*p* = 0.84), and hCD8^+^ (*p* = 0.71) T cells were similar between treatments (Figure 2B–D). Consequently, there was also no difference seen in the hCD4^+^:hCD8^+^ T cell ratio between treatments (*p* = 1.0) (Figure 2E). As expected, there was a significantly higher proportion of hTregs in hPBMCs incubated with the anti-hP2X7 mAb compared to those incubated with the isotype control mAb (*p* = 0.01) (Figure 2F). Furthermore, hPBMCs cultured with the anti-hP2X7 mAb showed a significantly larger proportion of hCD56^+^ hCD3^+^ NK T cells compared to cells incubated with the isotype control mAb (*p* = 0.04) (Figure 2G). Proportions of hCD56^+^ hCD3^−^ NK cells (*p* = 0.17) (Figure 2H) and hCD19^+^ B cells (*p* = 1.0) (Figure 2I) were similar between treatments.

### 3.3. The Anti-hP2X7 mAb Does Not Affect Early GVHD Development in Humanised Mice

**Figure 3:**
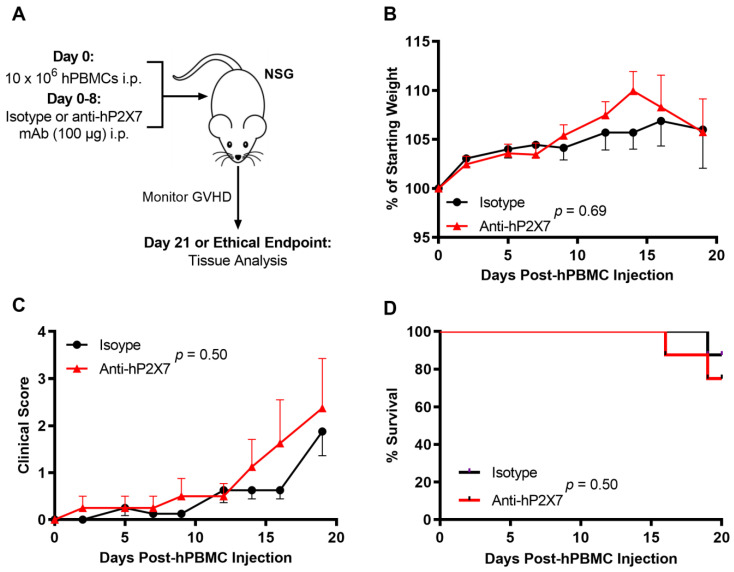
The anti-hP2X7 mAb did not affect early clinical GVHD development in humanised mice over 21 days. (**A**) Illustration of a humanised mouse model of GVHD. NSG mice were injected with 10 × 10^6^ hPBMCs (*n* = 2 donors) at Day 0 followed by 100 μg of the isotype control or anti-hP2X7 mAb every second day from Days 0–8 (*n* = 8 mice for each group). Mice were monitored thrice weekly over 21 days for (**B**) weight loss, (**C**) clinical score or (**D**) survival. (**B**,**C**) Data represented as mean ± SEM. Significance was assessed by the (**B**,**C**) two-way ANOVA with a Bonferroni post-test or (**D**) Mantel–Cox log-rank test for survival, with *p* values as shown.

To investigate the role of donor P2X7 in a humanised mouse model of GVHD, NSG mice were injected with 10 × 10^6^ hPBMCs at Day 0 followed by 100 μg of the anti-hP2X7 or isotype control mAb every second day from Days 0–8. Mice were monitored thrice weekly and euthanised at early stage GVHD (Day 21) (Figure 3A). As observed previously [20], clinical GVHD was minimal over 21 days in most mice with no significant difference in weight loss (*p* = 0.69) (Figure 3B), clinical score (*p* = 0.50) (Figure 3C), or survival (*p* = 0.50) (Figure 3D) between the anti-hP2X7 mAb and isotype control mAb-treated mice.

**Figure 4:**
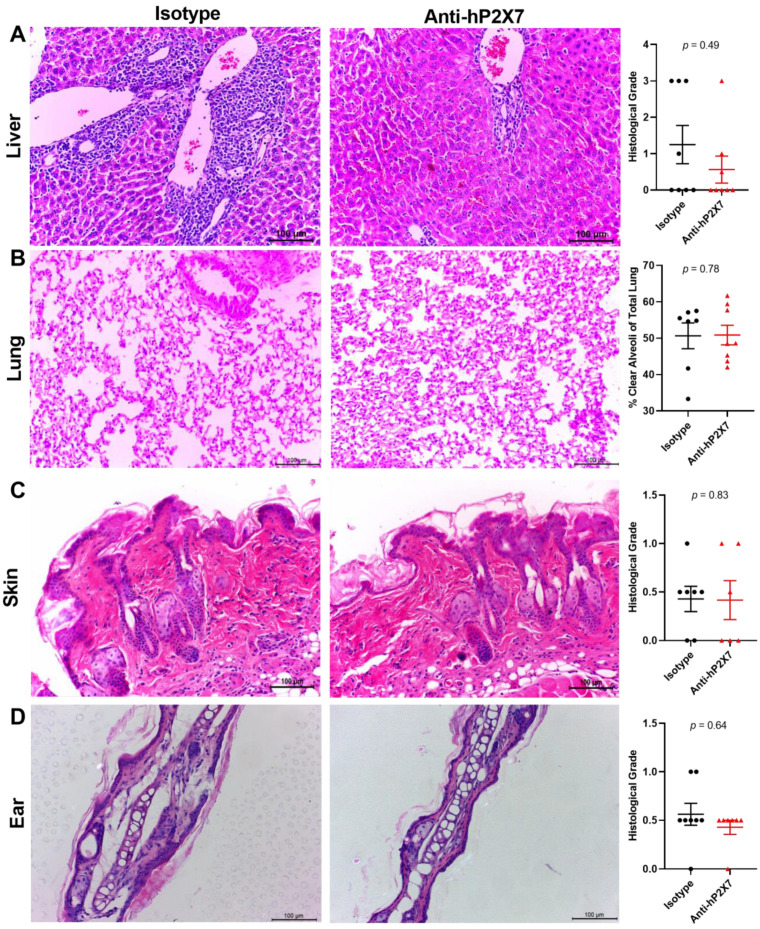
The anti-hP2X7 mAb does not affect histological GVHD in the liver, lung, skin, and ear of humanised mice at Day 21. The (**A**) liver, (**B**) lung, (**C**) skin, and (**D**) ear from isotype control (*n* = 7–8) or anti-hP2X7 (*n* = 6–8) mAb-treated mice at Day 21 (or humane endpoint) were sectioned (3–5 μm), stained, and graded based on evidence of histological GVHD. (**A**,**C**,**D**) Liver, skin, and ear were measured using a grading system. (**B**) Histological GVHD in the lung was measured as the percentage of clear alveoli space of total lung area. Images represent at least six mice per treatment group. (**A**–**D**) Scale bars represent 100 µm and data represented as the mean ± SEM. Symbols represent individual mice. Significance was assessed by the Mann–Whitney test, with *p* values as shown.

Previous studies utilising this humanised mouse model of GVHD have shown that the liver, lung, skin and ear are the main organs affected by leukocyte infiltration and tissue damage [20,21,22,30], with minimal histological GVHD in most organs except the liver at earlier time points [21]. Livers from mice treated with the isotype control mAb showed a 2.2-fold increase in signs of leukocyte infiltration and tissue damage compared to the livers from the anti-hP2X7 mAb-treated mice, but this increase did not reach statistical significance (*p* = 0.49) (Figure 4A). The percentage of clear alveoli space was near-normal and similar in both treatment groups (*p* = 0.78) (Figure 4B). Likewise, the amount of leukocyte infiltration and tissue damage in the skin (*p* = 0.83) (Figure 4C) and ear (*p* = 0.64) (Figure 4D) were negligible and similar between both treatment groups.

### 3.4. The Anti-hP2X7 mAb Increases Proportions of Splenic hTregs and hNK Cells at Day 21

**Figure 5:**
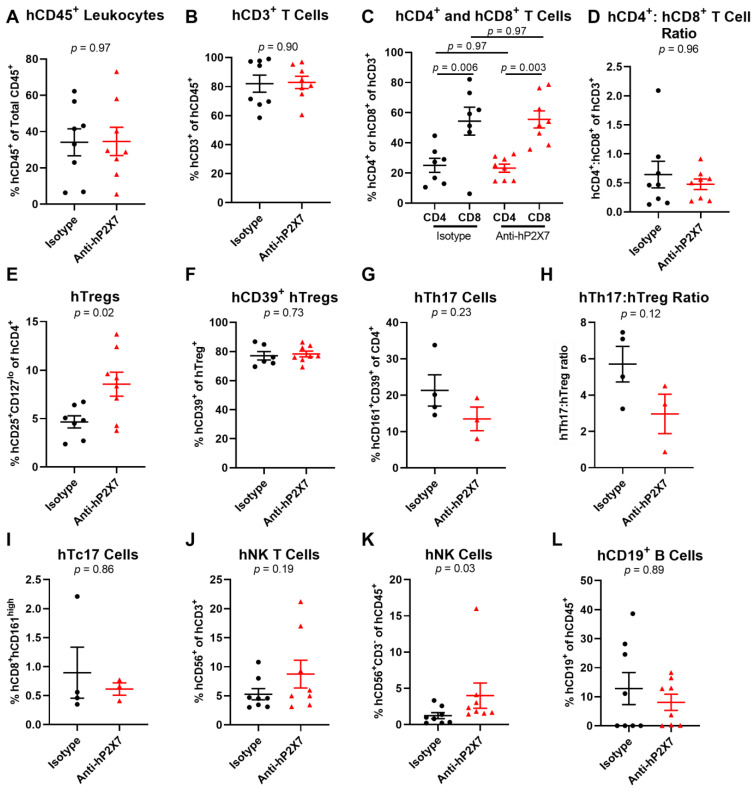
The anti-hP2X7 mAb increases proportions of splenic hTregs and hNK cells in humanised mice at Day 21. (**A**–**L**) Spleens from mice treated with the isotype control (*n* = 8) or anti-hP2X7 (*n* = 8) mAb were collected at Day 21 (or humane endpoint) and immune cell subsets were analysed by flow cytometry. Proportions of (**A**) hCD45^+^ leukocytes were first identified before determining the proportions of (**B**) hCD3^+^ T cells, (**C**) hCD4^+^ and hCD8^+^ T cells, (**E**) hCD4^+^hCD25^+^hCD127^lo^ hTregs, (**F**) hCD39^+^ hTregs, (**G**) hCD4^+^hCD161^+^hCD39^+^ hTh17 cells, (**I**) hCD8^+^hCD161^high^ Tc17 cells, (**J**) hCD3^+^hCD56^+^ hNK T cells, (**K**) hCD3^−^hCD56^+^ hNK cells, and (**L**) hCD19^+^ B cells. (**D**) The hCD4^+^:hCD8^+^ T cell ratio was calculated from **(C)**. (**H**) The hTh17:hTreg ratio was calculated from (**E**,**G**). (**A**–**L**) Data represented as the mean ± SEM. Symbols represent individual mice. Significance was assessed by the (**A**,**B**,**E**–**H**) unpaired Student’s *t*-test, (**C**) one-way ANOVA, or (**D**,**I**–**L**) Mann–Whitney test, with *p* values as shown.

To determine if the anti-hP2X7 mAb had an effect on the human leukocyte proportions at Day 21, spleens and livers were assessed by flow cytometry using the consistent gating strategy shown in Figure S2. Analysis of the spleens showed that the proportions of hCD45^+^ leukocytes (*p* = 0.97) (Figure 5A) and hCD3^+^ T cells (*p* = 0.90) (Figure 5B) were comparable between groups. Proportions of hCD4^+^ (*p* = 0.97) or hCD8^+^ (*p* = 0.97) T cells were also alike between both treatment groups (Figure 5C), which resulted in similar hCD4^+^:hCD8^+^ T cell ratios (*p* = 0.96) (Figure 5D). In contrast, the proportion of hTregs was significantly greater in the anti-hP2X7 mAb-treated mice compared to the isotype control mAb-treated mice (*p* = 0.02) (Figure 5E). Conversely, there was no difference in the highly suppressive hCD39^+^ hTregs subset (*p* = 0.73) (Figure 5F). The proportion of hTh17 cells was reduced by 36.7% in the anti-hP2X7 mAb-treated mice compared to the isotype control mAb-treated mice but this did not reach statistical significance (*p* = 0.23) (Figure 5G). Likewise, the hTh17:hTreg ratio was reduced by 48% in the anti-hP2X7 mAb-treated mice compared to the isotype control mAb-treated mice (*p* = 0.12) (Figure 5H). The proportions of hTc17 (*p* = 0.86) (Figure 5I), hNK T cells (*p* = 0.19) (Figure 5J), and B cells (*p* = 0.89) (Figure 5L) were similar between treatment groups. Conversely, the proportions of hNK cells were significantly greater in the anti-hP2X7 mAb-treated mice compared to the isotype control mAb-treated mice (*p* = 0.03) (Figure 5K). 

The proportions of the above cell types including hTregs and hNK cells in the liver were similar between treatment groups; hCD45^+^ leukocytes (*p* = 0.67) (Figure S3A), hCD3^+^ (*p* = 0.62) (Figure S3B), hCD4^+^ (*p* = 0.99) (Figure S3C) and hCD8^+^ (*p* = 0.65) (Figure S3C) T cells, the hCD4^+^:hCD8^+^ T cell ratio (*p* = 0.36) (Figure S3D), hTregs (*p* = 0.34) (Figure S3E), hNK cells (*p* = 0.77) (Figure S3K), hCD39^+^ hTregs (*p* = 0.69) (Figure S3F), hTh17 (*p* = 0.24) (Figure S3G), hTh17:hTreg ratio (*p* = 0.63) (Figure S3H), hTc17 (*p* = 0.36) (Figure S3I), hNK T cells (*p* = 0.78) (Figure S3J), and B cells (*p* = 0.38) (Figure S3L).

### 3.5. The Anti-hP2X7 mAb Does Not Affect Human Cytokine Concentrations in Sera at Day 21

Cytokines play significant roles in promoting GVHD progression [4]. Thus, to determine whether human cytokine concentrations were altered in the sera of mice between treatment groups, a human T Helper-1 LEGENDplex Kit was used to analyse 13 different cytokines. Serum hIL-5 was 5-fold greater in the anti-hP2X7 mAb-treated mice (56.15 pg/mL) compared to the isotype control mAb-treated mice (11.61 pg/mL), but this increase was not statistically significant (*p* = 0.19) (Figure S4B). Serum hIL-6 (*p* = 0.66) (Figure S4C) and hIL-9 (*p* = 0.43) (Figure S4D) concentrations were also slightly increased 1.7-fold and 1.1-fold, respectively, in the anti-hP2X7 mAb-treated mice compared to the isotype control mAb-treated mice but again did not reach statistical significance. Serum hIFNγ concentrations were also similar between treatment groups (*p* = 0.89) (Figure S4F). Serum hIL-2, hIL-10, hIL-13, hIL-22, and human tumour necrosis factor (hTNF) α all showed no significant differences between the anti-hP2X7 and isotype control mAb-treated mice (*p* = 1.0) (Figure S4A,E,G–I). Serum hIL-4, hIL-17A, hIL-17F, and hIL-21 were also assessed but were below the detection threshold.

### 3.6. The Anti-hP2X7 mAb Reduces Clinical GVHD in Humanised Mice

**Figure 6:**
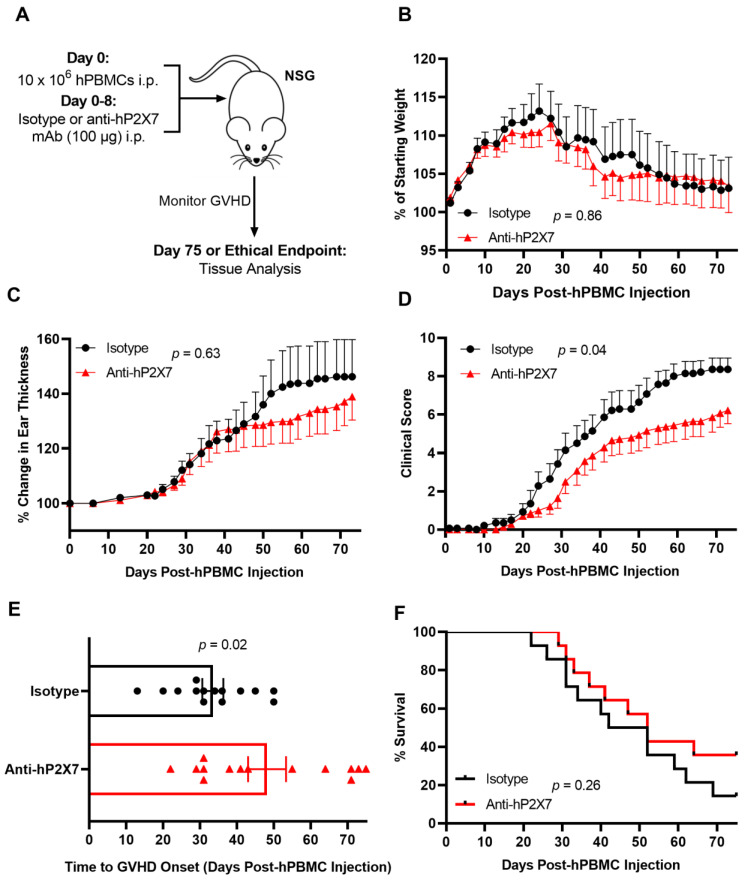
The anti-hP2X7 mAb reduces clinical GVHD in humanised mice. (**A**) Illustration of the humanised mouse model of GVHD. NSG mice were injected with 10 × 10^6^ hPBMCs (*n* = 4 donors) at Day 0 followed by 100 μg of isotype control or anti-hP2X7 (*n* = 14 mice for each group) mAb every second day for 8 days. Mice were monitored three times weekly for 75 days for (**B**) weight loss, (**C**) ear thickness, (**D**) clinical score, (**E**) time to GVHD onset, and (**F**) survival. Data represented as the mean ± SEM. (**E**) Symbols represent individual mice. Significance was assessed by the (**B**–**D**) two-way ANOVA with a Bonferroni post-test, (**E**) unpaired Student’s *t*-test, or (**F**) Mantel–Cox log-rank test for survival, with *p* values as shown.

To further investigate the role of donor P2X7 in GVHD, humanised mice were treated as above and monitored to humane (disease) or experimental endpoint (Day 75) (Figure 6A). Similarly to the Day 21 model, no significant difference was seen in weight loss between treatment groups (*p* = 0.86) (Figure 6B). Recently, ear thickness was established as a sign of cutaneous GVHD in this humanised mouse model [30]. The current study showed no significant difference in ear thickness between the anti-hP2X7 mAb-treated mice compared to the isotype control mAb-treated mice (*p* = 0.63) (Figure 6C). Notably, the anti-hP2X7 mAb-treated mice showed a significant reduction in clinical score compared to the isotype control mAb-treated mice (*p* = 0.04) (Figure 6D). Time to GVHD onset, defined as the day a mouse reaches a clinical score total of three or more [33], was also significantly delayed in the anti-hP2X7 mAb-treated mice compared to the isotype control mAb-treated mice (*p* = 0.02) (Figure 6E). However, differences in clinical score and disease onset did not result in increased survival in the anti-hP2X7 mAb-treated mice compared to the isotype control mAb-treated mice (*p* = 0.26) (Figure 6F).

### 3.7. The Anti-hP2X7 mAb Reduces Histological GVHD in the Liver and Lung at Endpoint

**Figure 7:**
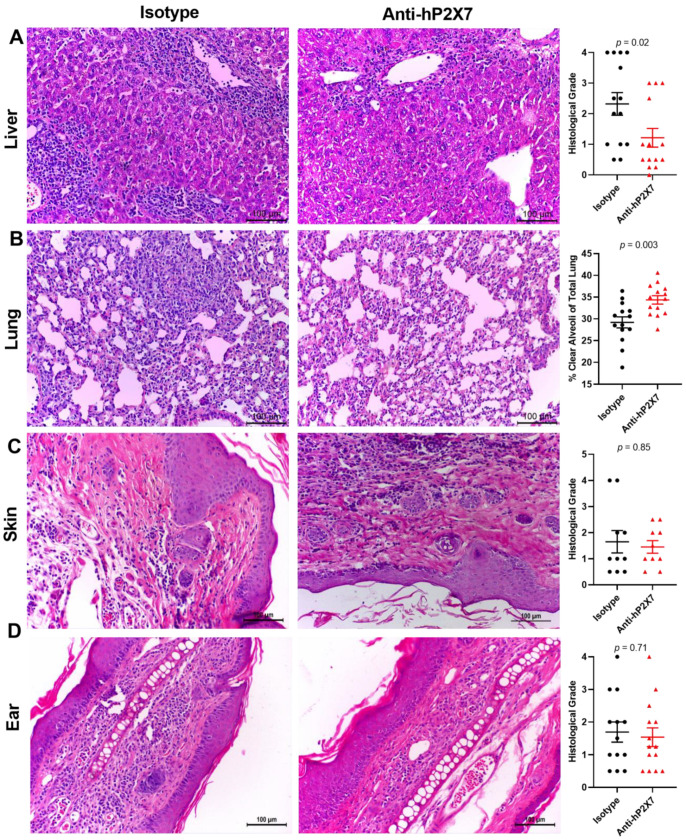
The anti-hP2X7 mAb reduces histological GVHD in the liver and lung at humane or experimental (Day 75) endpoint. The (**A**) liver, (**B**) lung, (**C**) skin, and (**D**) ear from the isotype control (*n* = 10–14) or anti-hP2X7 (*n* = 10–14) mAb-treated mice at endpoint were sectioned (3–5 μm), stained, and graded based on evidence of histological GVHD. (**A**,**C**,**D**) Liver, skin, and ear were measured using a grading system. (**B**) Histological GVHD in the lung was measured as the percentage of clear alveoli space of the total lung area. Images representative of at least 10 mice per treatment group. (**A**–**D**) Scale bars represent 100 µm. Data represented as the mean ± SEM. Symbols represent individual mice. Significance was assessed by the (**A**) unpaired Student’s *t*-test or (**B**–**D**) Mann–Whitney test, with *p* values as shown.

Histological GVHD was assessed in the target organs of the mice at humane or experimental (Day 75) endpoint. Livers from mice treated with the anti-hP2X7 mAb showed reduced leukocyte infiltration and tissue damage compared to the isotype control mAb-treated mice. This difference resulted in a significant reduction in histological grade in the anti-hP2X7 mAb-treated mice compared to the isotype control mAb-treated mice (*p* = 0.02) (Figure 7A). Moreover, lung pathology was reduced, with the percentage of clear alveoli space significantly increasing in the anti-hP2X7 mAb-treated mice compared to the isotype control mAb-treated mice (*p* = 0.002) (Figure 7B). In contrast, the skin and ear pathology were similar between treatment groups, with the mice each displaying comparable levels of epidermal thickening and immune cell infiltration, with no significant difference in histological grade in the flank skin (*p* = 0.85) (Figure 7C) or ears (*p* = 0.89) (Figure 7D) of the mice, paralleling similarities in ear thickness between the groups above (Figure 6C). 

### 3.8. The Anti-hP2X7 mAb Altered Cell Proportions in the Spleen and Liver of Humanised Mice at Endpoint 

**Figure 8:**
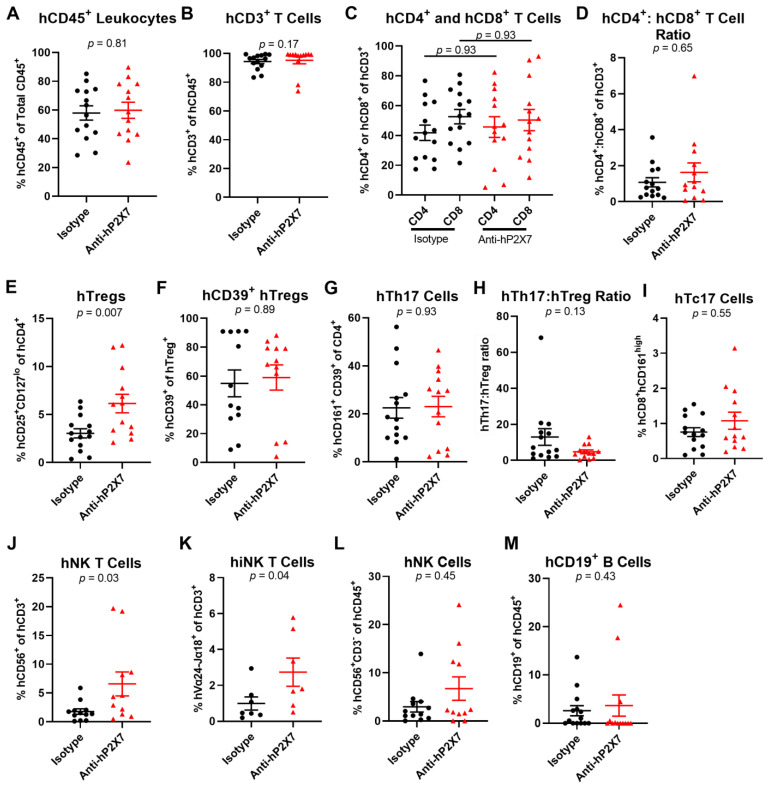
The anti-hP2X7 mAb altered cell proportions in the spleen of humanised mice at humane or experimental (Day 75) endpoint. (**A**–**M**) Spleens from mice treated with isotype control (*n* = 7–14) or anti-hP2X7 (*n* = 7–13) mAb were collected at humane or experimental (Day 75) endpoint and immune cell subsets were analysed by flow cytometry. Proportions of (**A**) hCD45^+^ leukocytes were first identified before determining proportions of (**B**) hCD3^+^ T cells, (**C**) hCD4^+^ and hCD8^+^ T cells, (**E**) hCD4^+^hCD25^+^hCD127^lo^ hTregs, (**F**) hCD39^+^ hTregs, (**G**) hCD4^+^hCD161^+^hCD39^+^ hTh17 cells, (**I**) hCD8^+^hCD161^high^ hTc17 cells, (**J**) hCD3^+^hCD56^+^ hNK T cells, (**K**) hCD3^+^ hVα24-Jα18^+^ hiNK T cells, (**L**) hCD3^−^hCD56^+^ hNK cells and (**M**) hCD19^+^ B cells. (**D**) The hCD4^+^:hCD8^+^ T cell ratio was calculated from (**C**). (**H**) The hTh17:hTreg ratio was calculated from (**E**,**G**). (**A**–**M**) Data represented as mean ± SEM. Symbols represent individual mice. Significance was assessed by the (**A**,**E**,**G**) unpaired Student’s *t*-test, (**B**,**D**,**F**,**H**–**M**) Mann-Whitney test, or (**C**) one-way ANOVA, with *p* values as shown.

Splenic and liver human leukocyte proportions in the mice were analysed at humane or experimental (Day 75) endpoint. In the spleen, flow cytometric analysis showed that mice treated with the anti-hP2X7 mAb, compared to those treated with the isotype control mAb, did not alter the proportions of the hCD45^+^ leukocytes (*p* = 0.81) (Figure 8A), hCD3^+^ T cells (*p* = 0.17) (Figure 8B), and hCD4^+^ (*p* = 0.93) (Figure 8C) and hCD8^+^ (*p* = 0.93) (Figure 8C) T cells. The hCD4^+^:hCD8^+^ T cell ratio *(p* = 0.65) was similar between treatment groups (Figure 8D). As observed in the short-term model (Figure 5E), the proportion of hTregs was significantly increased in the anti-hP2X7 mAb-treated mice compared to the isotype control mAb-treated mice (*p* = 0.007) (Figure 8E), but the proportions of hCD39^+^ hTregs were similar between treatment groups (*p* = 0.89) (Figure 8F). The proportion of hTh17 cells was similar across both groups (*p* = 0.93) (Figure 8G), however, the hTh17:hTreg ratio was reduced by 63.5% in the anti-hP2X7 mAb-treated mice compared to the isotype control mAb-treated mice, but this did not reach significance (*p* = 0.13) (Figure 8H). The proportion of hTc17 cells was alike in both groups (*p* = 0.55) (Figure 8I). Proportions of hCD56^+^ hCD3^+^ cells (*p* = 0.03) (Figure 8J), classified as hNK T cells, and hVα24-Jα18^+^ hCD3^+^ (*p* = 0.04) (Figure 8J), classified as invariant (i) NK T cells were significantly increased in the anti-hP2X7 mAb-treated mice compared to the isotype control mAb-treated mice. Proportions of hCD56^+^ hCD3^−^ hNK cells (*p* = 0.45) (Figure 8L) and hCD19^+^ B cells (*p* = 0.43) (Figure 8M) were alike between both treatment groups.

**Figure 9:**
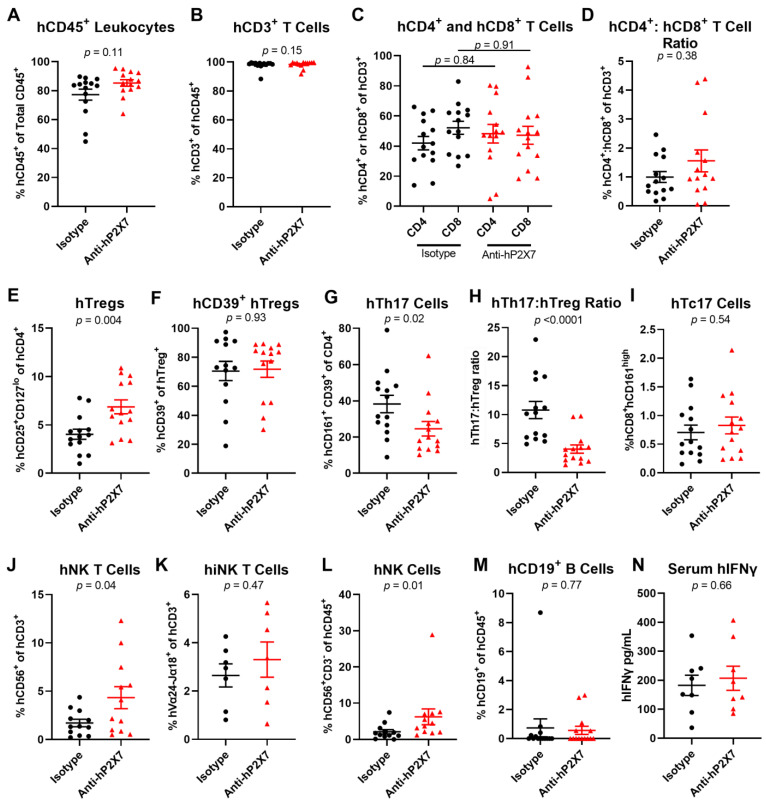
The anti-hP2X7 mAb altered the cell proportions in the liver of humanised mice at endpoint. (**A**–**M**) Livers from mice treated with isotype control or anti-hP2X7 (*n* = 7–14) mAb were collected at humane or experimental (Day 75) endpoint and immune cell subsets were analysed by flow cytometry. Proportions of (**A**) hCD45^+^ leukocytes were first identified before determining the proportions of (**B**) hCD3^+^ T cells, (**C**) hCD4^+^ and hCD8^+^ T cells, (**E**) hCD4^+^hCD25^+^hCD127^lo^ hTregs, (**F**) hCD39^+^ hTregs, (**G**) hCD4^+^hCD161^+^hCD39^+^ hTh17 cells, (**I**) hCD8^+^hCD161^high^ hTc17 cells, (**J**) hCD3^+^hCD56^+^ hNK T cells, (**K**) hCD3^+^ hVα24-Jα18^+^ hiNK T cells, (**L**) hCD3^−^hCD56^+^ hNK cells, and (**M**) hCD19^+^ B cells. (**D**) The hCD4^+^:hCD8^+^ T cell ratio was calculated from (**C**). (**H**) The hTh17:hTreg ratio was calculated from (**E**,**G**). (**N**) hIFNγ was assessed by ELISA in serum collected from the humanised NSG mice that were treated with the isotype control or anti-hP2X7 (*n* = 8) mAb. Data represented as the mean ± SEM. Symbols represent individual mice. Significance was assessed by (**A**,**B**,**D**,**F**–**H**,**L**,**M**) the Mann–Whitney test, (**C**) one-way ANOVA, or (**E**,**I**,**J**,**K**,**N**) unpaired Student’s *t*-test, with *p* values as shown.

In the liver, proportions of hCD45^+^ leukocytes (*p* = 0.11) (Figure 9A), hCD3^+^ T cells (*p* = 0.15) (Figure 9B), hCD4^+^ (*p* = 0.84) (Figure 9C) and hCD8^+^ T cells (*p* = 0.91) (Figure 9C), and the hCD4^+^:hCD8^+^ T cell ratio (*p* = 0.38) (Figure 9D) were alike between the treatment groups. The proportion of hTregs was significantly increased in the anti-hP2X7 mAb-treated mice compared to the isotype control mAb-treated mice (*p* = 0.004) (Figure 9E), however, the proportion of hCD39^+^ hTregs was similar between treatment groups (*p* = 0.93) (Figure 9F). Notably, the hTh17 cells were significantly decreased in the anti-hP2X7 mAb-treated mice compared to the isotype control mAb-treated mice (*p* = 0.02) (Figure 9G). This resulted in a significant decrease in the hTh17:hTreg ratio in the anti-hP2X7 mAb-treated group compared to the isotype control mAb-treated group (*p* < 0.0001) (Figure 9H). Proportions of hCD56^+^ hCD3^+^ hNK T cells (*p* = 0.04) (Figure 9J) and hCD56^+^ hCD3^+^ hNK cells (*p* = 0.01) (Figure 9L) were significantly increased in the anti-hP2X7 mAb-treated mice compared to the isotype control mAb-treated mice. However, the proportion of hVα24-Jα18^+^ hCD3^+^ hiNK T cells was not different between treatment groups (*p* = 0.47) (Figure 9K). Proportions of hTc17 cells (*p* = 0.54) (Figure 9I) and hCD19^+^ B cells (*p* = 0.77) (Figure 9M) were also alike between treatments.

IFNγ is a cytokine known to be involved in the progression of GVHD. Previous P2X7 blockade studies in this model have shown a reduced serum hIFNγ concentration in humanised mice at endpoint [20,21]. Therefore, this cytokine in sera was measured in mice at endpoint by ELISA. No significant difference was seen in sera hIFNγ concentrations between the anti-hP2X7 mAb-treated mice and isotype control mAb-treated mice (*p* = 0.66) (Figure 9N). 

## 4. Discussion

Through the use of a species-specific anti-hP2X7 mAb, the current study aimed to investigate whether donor P2X7 contributes to GVHD development in a humanised mouse model. The ability of the anti-hP2X7 mAb to bind and block human but not mouse P2X7, as previously demonstrated with P2X7-transfected cells [24], was confirmed using in vitro studies involving human RPMI 8226 and murine J774 cells. Further in vitro studies using serum starved hPBMCs demonstrated that the anti-hP2X7 mAb could prevent the loss of hTregs and hNK T cells. Most notably, the anti-hP2X7 mAb reduced clinical and histological GVHD in a humanised mouse model, which corresponded to an increase in the spleen and liver hTregs and hNK T cells and liver hNK cells, and a decrease in the liver hTh17 cells, resulting in a reduced hTh17:hTreg ratio.

The current study provides the first direct evidence that donor (human) P2X7 contributes directly to GVHD progression in any mouse model of this disease. Administration of the small molecule P2X7 antagonist, BBG, in the same humanised mouse model as used here, revealed that P2X7 contributes to clinical and histological GVHD [20,21,22] but whether donor or host P2X7 or both species of this receptor contributed to GVHD progression could not be determined with this antagonist, which impairs both human and murine P2X7 [34]. However, the comparison of donor hPBMCs coding for gain- or loss-of-function *P2RX7* gene polymorphisms in this model originally suggested that donor P2X7 does not contribute to disease progression [35]. Moreover, use of the small molecule P2X7 antagonist, AZ10606120, which impairs both human and murine P2X7 [36], did not alter GVHD progression in this same humanised mouse model [37], raising the possibility that P2X7 was not involved and that BBG in previous studies [20,21,22] may have acted via another target. Thus, the use of the anti-hP2X7 mAb in the current study establishes a role for donor (human) P2X7 in GVHD progression in humanised mice. Reasons for the differences observed with the anti-hP2X7 mAb versus donor hPBMCs with *P2RX7* gene polymorphisms may be explained by the potentially greater blockade of P2X7 activity by the mAb versus the incomplete loss of P2X7 activity in donors who were heterozygous for a single loss-of-function polymorphism [35], while the observed differences in GVHD between the anti-hP2X7 mAb versus AZ10606120 suggest that the AZ10606120 regimen used was insufficient to block P2X7 activity in vivo. Whether host (murine) P2X7 contributes to GVHD development in humanised mice remains unknown, but a study of bone marrow chimeras using *P2rx7* gene knockout and wild-type mice indicated that P2X7 on host APCs but not donor cells was the main contributor to GVHD development in an allogeneic mouse model [16]. Therefore, further studies are required using a species-specific mP2X7 antibody or nanobody [38] to determine a role for host P2X7 in GVHD progression in humanised mice.

Immune cell profiling revealed that the reduction in clinical and histological GVHD in humanised mouse by the anti-hP2X7 mAb corresponded to an increase in hTregs and to a lesser extent hNK T cells and hNK cells and a reduction in hTh17 cells, which also corresponded to a decreased hTh17:hTreg ratio. These results largely parallel observations in humans following allogeneic HSCT, where higher proportions of hTregs, hNK T cells, and hNK cells correlate with decreased GVHD severity [39,40,41], and where increased proportions of hTh17 cells or higher ratios of hTh17:hTreg correlate with increased GVHD severity [42,43]. Moreover, hTregs and hNK T cells have been shown to reduce GVHD severity in humanised NSG mice [44,45], while hNK cells are required for intravenous immunoglobulin-induced GVHD prevention in these mice [46]. In contrast, hTh17 cells can worsen GVHD in these mice [47]. Thus, it is likely that the anti-hP2X7 mAb reduced GVHD progression by altering these human lymphocyte subsets. Consistent with this, previous studies using small molecule P2X7 antagonists in allogeneic [16] or humanised [21] mice to prevent GVHD corresponds to an increase in donor Tregs. Whether P2X7 blockade alters Treg quiescence, differentiation, and exhaustion [48] remains to be determined.

In vitro studies revealed that the anti-hP2X7 mAb prevents the loss of hTregs and hNK T cells during serum starvation, suggesting that this mAb may also act to prevent P2X7-mediated cell death, a process previously observed to occur in these cell types [12,15,49], to subsequently promote hTreg and hNK T cell survival and reduce GVHD progression in humanised mice. However, P2X7 blockade can also promote and prevent naïve CD4^+^ T cell differentiation to Tregs and Th17 cells, respectively [15], offering an alternative or additional explanation for the beneficial effects of the anti-hP2X7 mAb observed in vivo. This notion is supported by the observed decrease in the hTh17:hTreg ratio in humanised mice treated with the anti-hP2X7 mAb. Moreover, given that P2X7 activation on DCs can promote hTh17 cell differentiation [15,50,51], the possibility remains that the anti-hP2X7 mAb may prevent P2X7 activation on hDCs, if present following hPBMC injection, to impair hTh17 cell differentiation, leading to reduced proportions of these cells in vivo. Finally, the loss of P2X7 can prevent the pro-inflammatory effects of primed NK T cells [52], suggesting that in addition to promoting NK T cell survival, the anti-hP2X7 mAb may be preventing the activation of primed NK T cells in vivo. However, to the best of our knowledge, the priming of NK T cells during GVHD development in humanised NSG mice has not been reported.

In contrast to hTregs and hNK T cells, in vitro studies have revealed that the anti-hP2X7 mAb does not prevent the loss of hNK cells during serum starvation. Moreover, the role of P2X7 on NK cells continues to be undetermined. Thus, it remains unknown as to why the anti-hP2X7 mAb increased the proportions of these cells in humanised mice. Notably, this effect was not observed with BBG or with a second small molecule P2X7 antagonist, pyridoxalphosphate-6-axophenyl-2′-4′-disulfonic acid [53], in humanised mice [21], suggesting that the anti-hP2X7 mAb has greater efficacy on hNK cells than either small molecule P2X7 antagonists in vivo. Consistent with this and as discussed above, this mAb also altered the proportions of hTregs, hNK T cells, and hTh17 cells, and so collectively, a greater number of lymphocyte subsets are impacted by the anti-hP2X7 mAb than by any of the small molecule P2X7 antagonists used previously in this model [20,21,22,37]. Of note, it has been observed that P2X7 activation impairs the antitumour activity of NK cells to human chronic myeloid leukaemia K562 cells [54]. Thus, combined with the notion that NK cells may help to both prevent GVHD and contribute to graft-versus-leukaemia immunity [55], use of an anti-hP2X7 mAb in such recipients may help improve outcomes in people with leukaemia following allogeneic HSCT.

In contrast to the observed effects on immune cell proportions, the anti-hP2X7 mAb did not alter the circulating hIFNγ concentrations in humanised mice. In comparison, the non-species selective P2X7 antagonist, BBG, could reduce the circulating hIFNγ concentrations in these mice [20,21]. This suggests that the host, but not donor, P2X7, plays a role in hIFNγ production in this model of GVHD. If so, this implies that P2X7 activation on a mouse (host) cell can stimulate human (donor) immune cells to produce this cytokine and provides evidence of species crosstalk in these humanised mice. However, off-target effects of BBG, particularly blockade of pannexin-1 and the subsequent release of ATP from cells [11], cannot be excluded.

Finally, the current study provides the first report of an anti-hP2X7 biologic with inhibitory activity applied in any physiological or pathophysiological context in vivo. Previous applications of P2X7 biologics with inhibitory activity in vivo have been limited to the use of an anti-mP2X7 mAb (clone 1F11) to reduce colitis [23] and anti-mP2X7 nanobodies to ameliorate dermatitis or glomerulonephritis [56] and stroke lesions [57] in mice, while another study has demonstrated the use of capsid-modified adeno-associated viral vectors to deliver anti-mP2X7 nanobodies in vivo to reduce tumour growth in mice [58]. Combined, these studies support the future use of anti-hP2X7 biologics as potential therapeutics in people in which a role of P2X7 activation is indicated. The use of such biologics may help overcome some of the limitations observed with small molecule P2X7 antagonists in clinical trials, which to date have yielded unsatisfying results [59].

In conclusion, the current study confirmed the ability of an anti-hP2X7 mAb to bind and block human, but not mouse, P2X7. Moreover, this mAb could prevent the loss of hTregs and hNK T cells during serum starvation. Finally, the anti-hP2X7 mAb reduced clinical and histological GVHD in a humanised mouse model, which corresponded to increased proportions of hTregs, hNK T cells, and hNK cells and decreased the proportions of hTh17 cells and hTh17:hTreg ratio, collectively demonstrating that donor (human) P2X7 can directly contribute to GVHD progression. However, more research investigating this mAb including in combination with therapies targeting other GVHD mechanisms is required, for example, the use of the anti-P2X7 mAb with an anti-IFNγ or anti-IL-17 mAb.

## Data Availability

Data available from R.S. upon reasonable request.

## Supplementary Materials

The following supporting information can be downloaded at: https://www.mdpi.com/article/10.3390/pharmaceutics15092263/s1, Figure S1: Gating strategy used to identify human cell populations in vitro. Figure S2: Gating strategy used to identify human cell populations in vivo. Figure S3: The anti-hP2X7 mAb does not affect proportions of liver cell subsets in humanised mice at Day 21. Figure S4: The anti-hP2X7 mAb does not affect human cytokine concentrations in sera from humanised mice at Day 21. Table S1: Monoclonal antibodies used for the immunolabelling of immune cell subsets.
