# Supplementary material for: A Species-Specific Anti-Human P2X7 Monoclonal Antibody Reduces Graft-versus-Host Disease in Humanised Mice
Source: Pharmaceutics. 2023 Aug 31;15(9):2263. doi: 10.3390/pharmaceutics15092263 (PMC10536354; doi:10.3390/pharmaceutics15092263)
Supplement: Supplementary file 1 [file pharmaceutics-15-02263-s001.zip › pharmaceutics-2507375-supplementary.pdf]

pharmaceutics

MDPI

Supplementary Materials: A Species-Specific Anti-Human P2X7 Monoclonal Antibody Reduces Graft-Versus-Host Disease in Humanised Mice

Amal Elhage, Peter Cuthbertson, Chloe Sligar, Debbie Watson and Ronald Sluyter *

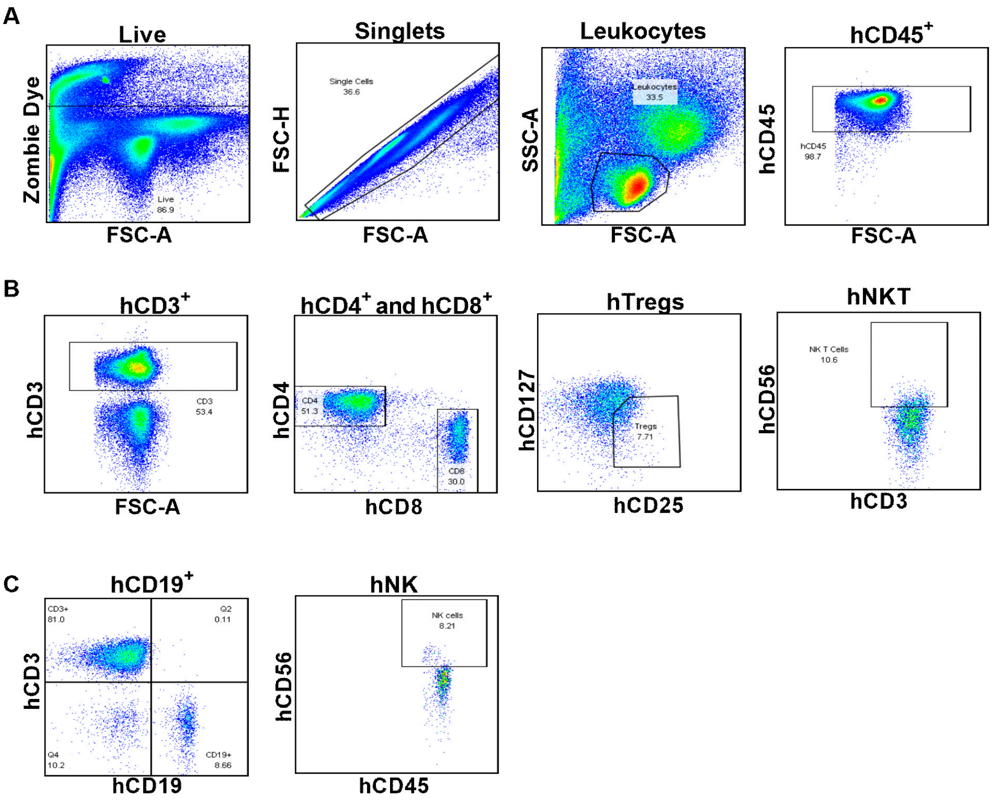

Figure S1: Gating strategy used to identify human cell populations in vitro. (**A-C**) Immune cell subsets were analysed by flow cytometry. (**A**) Live cells were gated based on Zombie NIR staining and forward scatter-area (FSC-A). Singlets were gated based on forward scatter-height (FSC-H) and FSC-A. Human leukocytes were gated using side scatter-area (SSC-A) and FSC-A. The proportion of $hCD45^+$ leukocytes was then identified before gating (**B**) $hCD3^+$ T cells, $hCD4^+$ and $hCD8^+$ T cell subsets, $hCD4^+hCD25^+hCD127^{lo}$ hTregs, $hCD3^+hCD56^+$ NK T cells, and (**C**) $hCD19^+$ B cells and $hCD3^-$ $hCD19^-$ $hCD56^+$ NK cells.

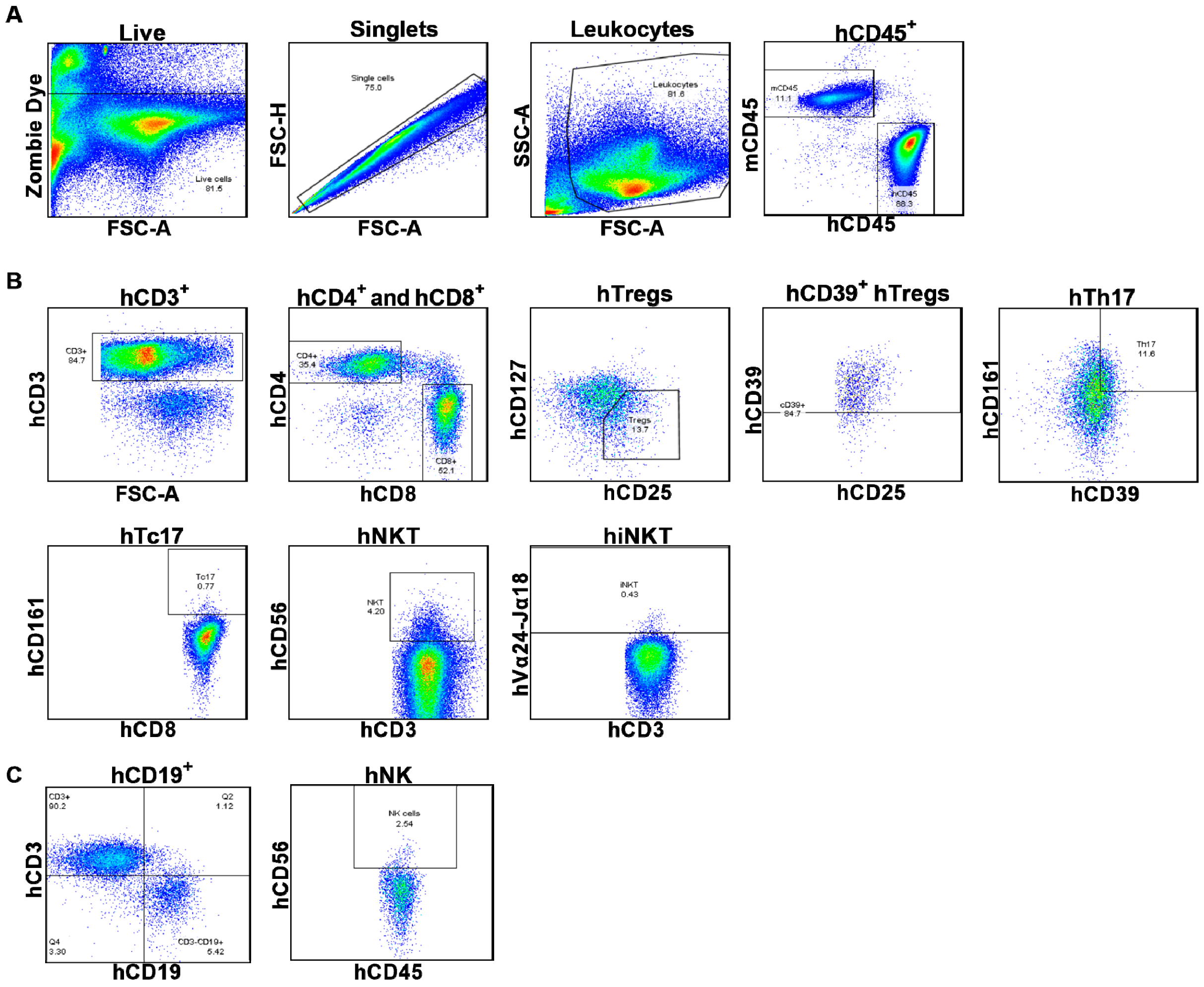

Figure S2: Gating strategy used to identify human cell populations in vivo. (**A-C**) Immune cell subsets were analysed by flow cytometry. (**A**) Live cells were gated based on Zombie NIR staining and forward scatter-area (FSC-A). Singlets were gated based on forward scatter-height (FSC-H) and FSC-A. Human leukocytes were gated using side scatter-area (SSC-A) and FSC-A. The proportion of $hCD45^{+}$ leukocytes was then identified before gating (**B**) $hCD3^{+}$ T cells, $hCD4^{+}$ and $hCD8^{+}$ T cell subsets, $hCD4^{+}hCD25^{+}hCD127^{lo}$ hTregs, $hCD39^{+}$ hTregs, $hCD4^{+}hCD161^{+}hCD39^{+}$ Th17 cells, $hCD8^{+}hCD161^{high}$ cells, $hCD3^{+}hCD56^{+}$ NK T cells, $hCD3^{+}hV\alpha24\text{-}J\alpha18^{+}$ iNKT cells, and (**C**) $hCD19^{+}$ B cells and $hCD3^{-}hCD19^{-}hCD56^{+}$ NK cells.

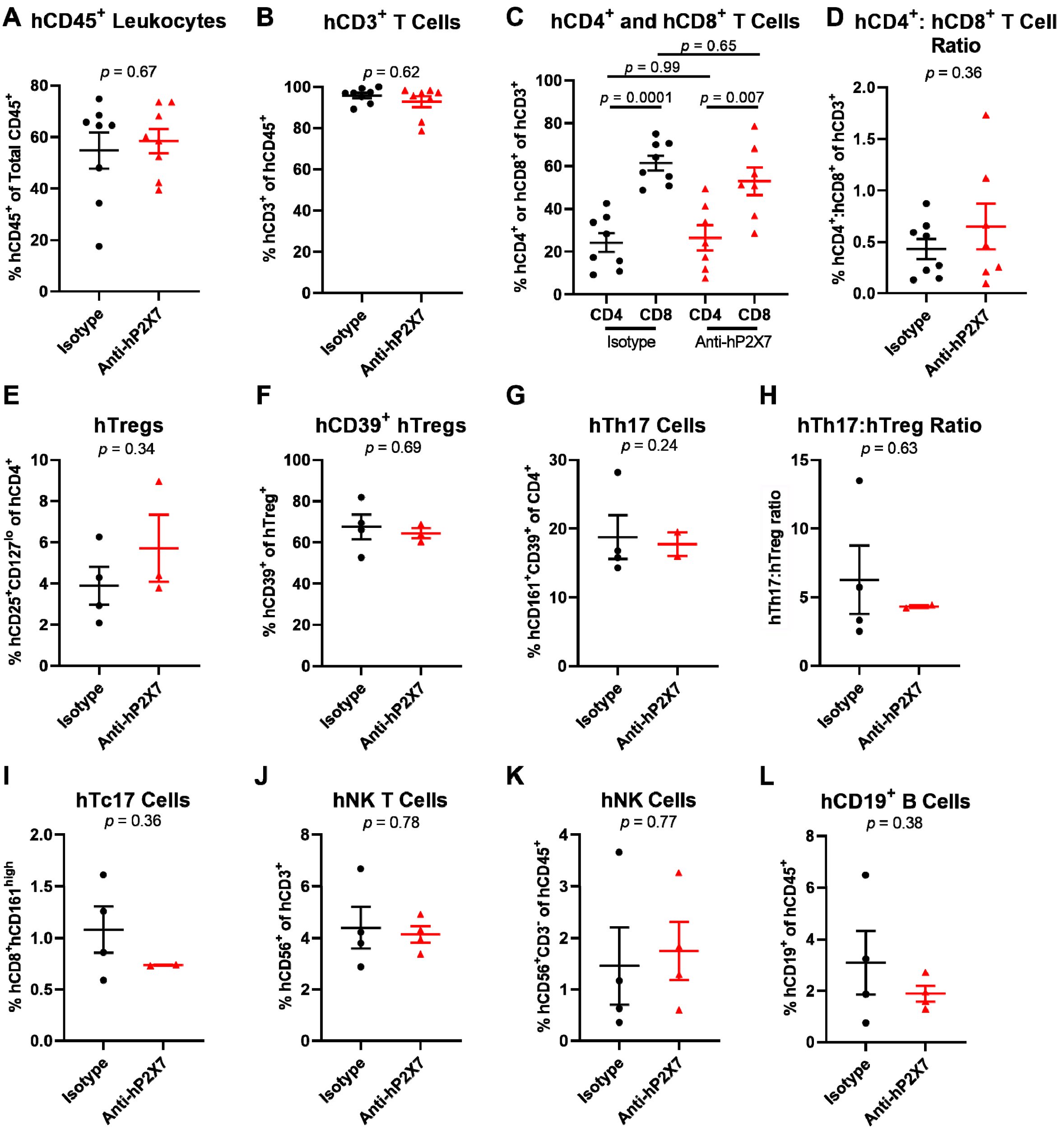

Figure S3: The anti-hP2X7 mAb does not affect proportions of liver cell subsets in humanised mice at Day 21. (**A-L**) Livers from mice treated with isotype control (n = 4-8) or anti-hP2X7 (n = 2-8) mAb were collected at Day 21 (or humane endpoint) and immune cell subsets were analysed by flow cytometry. Proportions of (**A**) $hCD45^+$ leukocytes were first identified before determining proportions of (**B**) $hCD3^+$ T cells, (**C**) $hCD4^+$ and $hCD8^+$ T cells, (**E**) $hCD4^+hCD25^+hCD127^{lo}$ hTregs, (**F**) $hCD39^+$ hTregs, (**G**) $hCD4^+hCD161^+hCD39^+$ hTh17 cells, (**I**) $hCD8^+hCD161^{high}$ hTc17 cells, (**J**) $hCD3^+hCD56^+$ hNK T cells, (**K**) $hCD3^-hCD56^+$ hNK cells and (**L**) $hCD19^+$ B cells. (**D**) The $hCD4^+$:$hCD8^+$ T cell ratio was calculated from (**C**). (**H**) The hTh17:hTreg ratio was calculated from (**E**) and (**G**). Data represented as mean ± SEM. Symbols represent individual mice. Significance was assessed by the (**A, D-L**) unpaired Student's t-test, (B) Mann-Whitney test or (**C**) one-way ANOVA, with p values as shown.

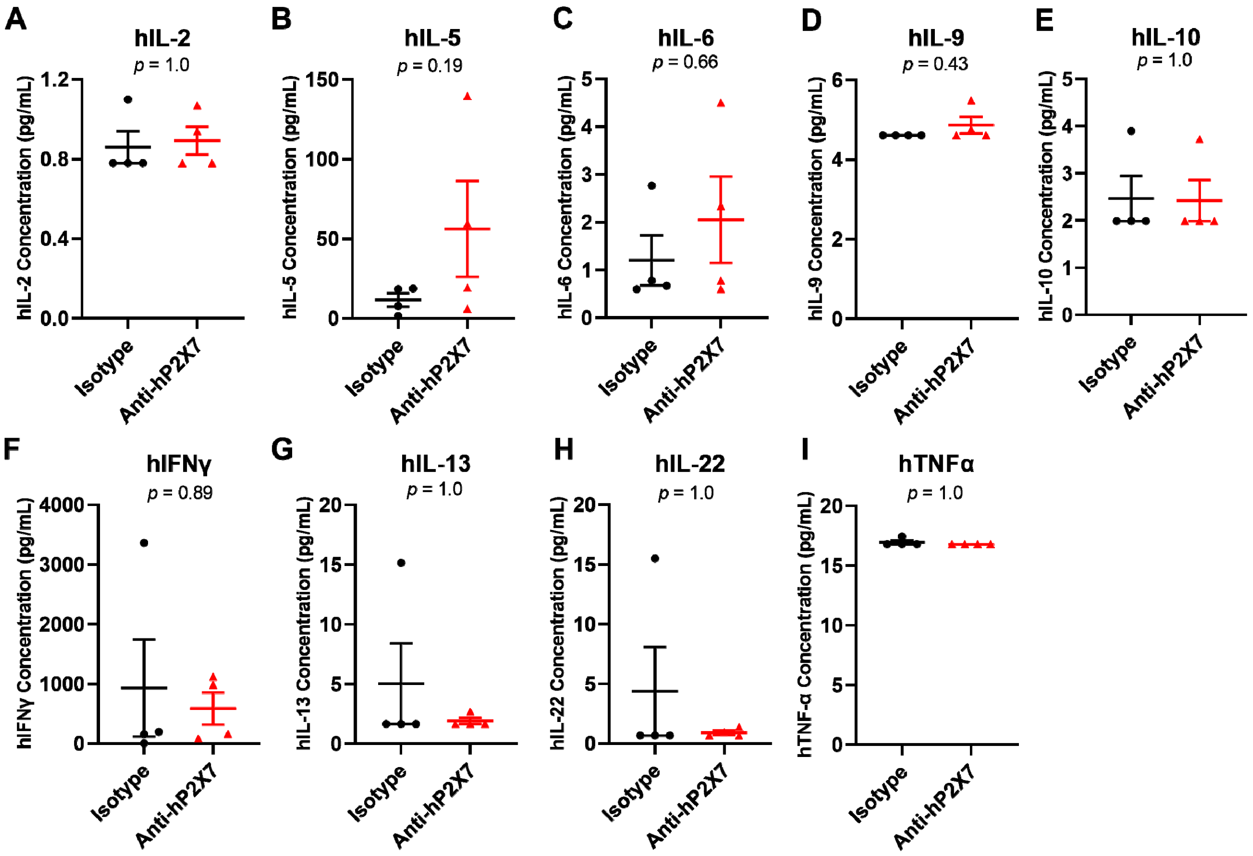

Figure S4: The anti-hP2X7 mAb does not affect human cytokine concentrations in sera from humanised mice at Day 21. (**A-I**) Serum from mice treated with the isotype control (n = 4) or anti-hP2X7 (n = 4) mAb were collected at Day 21 (or humane endpoint) and cytokine concentrations were analysed using a human T helper-1 LEGENDplex kit. Concentrations of serum human (**A**) IL-2, (**B**) IL-5, (**C**) IL-6, (**D**) IL-9, (**E**) IL-10, (**F**) IL-13, (**G**) IL-22, (**H**) TNFα and (**I**) IFNγ. Data represented as mean ± SEM. Symbols represent individual mice. Significance was assessed by the (**A, C-I**) Mann-Whitney test or (**B**) unpaired Student's t-test, with p values as shown.

Table S1: Monoclonal antibodies used for the immunolabelling of human cell subsets

**Antibodies**[a]	**Clone**	**Fluorochrome**[b]	**Dilutions**
CD3	UHCT1	BV711	1:50
CD4	RPA-T4	PerCP-Cy5.5	1:10
CD8	RPA-T8	PE-Cy7	1:50
CD19	HIB19	APC	1:20
CD25	M-A251	PE	1:20
CD39	TU66	APC	1:20
CD45	HI30	FITC	1:20
Anti-mouse CD45	30-F11	PerCP	1:20
CD56	MY31	PE	1:20
CD127	HIL-7R-M21	BV421	1:20
CD161	HP-3G10	BV605	1:50
Vα24-Jα18 (iNKT)	6B11	PE-Cy7	1:50

[a] All anti-human mAb except where indicated (BD Biosciences).
[b] BV; Brilliant violet, PerCP; peridinin chlorophyll protein-cyanine, PE-Cy; phycoerythrin-cyanine, APC; allophycocyanin and PE; phycoerythrin.
